# Thrombosis and lymphocyte subsets of COVID-19 omicron BA.2 variant patients with cancer

**DOI:** 10.3389/fonc.2022.1048999

**Published:** 2022-11-10

**Authors:** Jiaxin Yin, Xiaofeng Cong, Nanya Wang, Wei Song, Yanjie Guan, Yiqun Zhang, Zhi Li, Ziling Liu

**Affiliations:** Department of Oncology, The First Hospital of Jilin University, Changchun, Jilin, China

**Keywords:** COVID-19, cancer, deep vein thrombosis (DVT), lymphocyte subsets, IL-6

## Abstract

Severe acute respiratory syndrome coronavirus 2 (SARS-CoV-2) caused an ongoing global pandemic of COVID-19. It has been found that COVID-19 has an influence on the changes of blood coagulation parameters and the high incidence of thrombosis. Changchun experienced the epidemic of the Omicron BA.2 variant SARS-CoV-2 in March 2022 in China. Once infected, BA.2 spreads rapidly and most of them are asymptomatic. The purpose of this study is to research venous thrombosis and laboratory changes (including PLT, PT, APTT, DD, FDP, CRP, WBC, IL-6 and lymphocyte subsets) among 92 cancer patients with COVID-19 and 73 COVID-19 patients with non-cancer by Mann-Whitney U and Chi-square test. It was found that the levels of D-dimer, FDP, CRP and IL-6 in cancer patients were significantly higher than those in the COVID-19 cohort. There were 9 (9.8%) cancer patients and 2 (2.7%) non-cancer patients found VTE, with no significant difference. The results showed that WBC, lymphocytes and B cells in cancer patients were significantly lower than those in the other group. Prophylactic anticoagulation was recommended for cancer patients with high risk factors, while paying attention to the occurrence of bleeding events. The detection of leukocyte classification, D-dimer, prothrombin time and fibrinogen at different time points are helpful for the diagnosis and anticoagulation of COVID-19 patients with cancer.

## Introduction

Severe acute respiratory syndrome coronavirus 2 (SARS-CoV-2) caused an ongoing global pandemic of coronavirus disease 2019 (COVID-19), which have infected tens of millions of people (1). At present, it has been found that COVID-19 has an influence on the changes of blood coagulation parameters and the high incidence of thrombosis, which is related to the inflammatory response to SARS-CoV-2 (2–4). There are complex interactions among viruses, immune, coagulation pathways, as well as inflammatory reactions and endothelial injuries involved in microvascular and macrovascular thrombosis (2, 5). And cancer patients with blood hypercoagulable state, the incidence of venous thrombosis will be significantly increased (6, 7). However, there is no clear conclusion whether the probability of thrombosis will increase, as well as how to choose anticoagulation regimen in cancer patients infected with COVID-19 (8). The hospital set up COVID-19 cancer treatment center, and treated 92 patients with cancer and Omicron BA.2 variant COVID-19 systematically. Once infected, BA.2 spreads rapidly and most of them are asymptomatic. The purpose of this study is to research venous thrombosis, interleukin-6 (IL-6) and lymphocyte subsets among cancer patients with COVID-19 and COVID-19 patients with non-cancer. Herein, we summarize the numerous hematologic findings and thrombus of COVID-19 related to cancer and we provide guidance for early prevention and treatment of the latter.

## Methods

A retrospective analysis was performed of patients with a diagnosis of COVID-19 at the Bethune First Hospital of Jilin University, China, from April to May 2022. Inclusion required patients to be at least 18 years of age and to have a positive polymerase chain reaction test for SARS CoV-2 from pharyngeal swab.

The primary outcomes of interest were the cumulative incidences of thrombosis by colorful doppler ultrasound before leaving hospital. The secondary endpoints were lymphocyte subsets and IL-6 level. Data were collected by manual chart review of patients hospitalized with COVID-19 infection. Data were analyzed using SPSS 26.0. Baseline data included age, gender, platelets, D-dimer (DD), prothrombin time (PT), active partial thromboplasting time (APTT), Fibrin/Fibrinogen Degradation Products (FDP), C-reactive protein (CRP) and use of anticoagulant. The level of IL-6 was measure by ELISA. Because of the non-normal distribution of laboratory data in the study, Mann-Whitney U nonparametric test was used to analyze the significant difference between two independent cohorts. Chi-square test was used to analyze the difference in incidence rates, and correction test and pairwise comparison were noticed. All significance testing was two-sided with a *P* < 0.05 being indicative of statistical significance.

## Results

**Figure 1:**
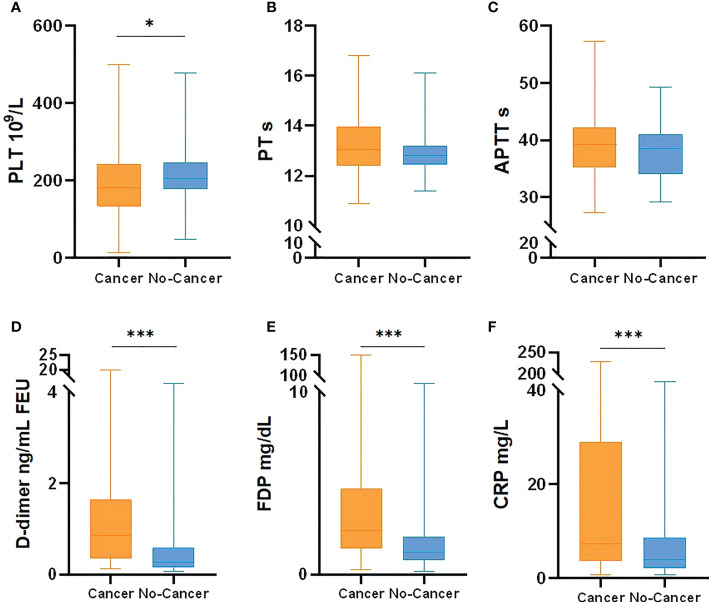
Coagulation related laboratory comparison between cancer patients with COVID-19 and no-cancer patients [**(A)** PLT, **(B)** PT, **(C)** APTT, **(D)** D-dimer, **(E)** FDP, **(F)** CRP]. *p < 0.05, ***p < 0.001.

The COVID-19 Cancer treatment Center was set up at the Bethune first Hospital of Jilin University in March 2022. A total of 92 cancer patients with COVID-19 met the criteria. At the same time, it also included data on 73 COVID-19 patients with non-cancer. The distribution of two cohorts was similar in age, sex and comorbidities (**Table 1**; **Figure 1**). Once BA.2 is infected, it spreads rapidly and most of them are asymptomatic.

**Table 1:** Baseline characteristics and VTE of cancer patients with COVID-19 and COVID-19 patients.

	Cancer+Covid-19 (N = 92)	Covid-19 (N = 73)	*P*
Age, median (IQR)	59.5 (50-66)	51 (42-59)	
Gender			
Female	51 (55.4%)	38 (52.1%)	
Male	41 (44.6%)	35 (47.9)	
Comorbidities, n (%)			
Hypertension	11 (11.96%)	9 (12.33%)	
Heart disease	8 (8.70%)	10 (13.70%)	
Diabetes	10 (10.87%)	1 (1.37%)	
Cerebralvascular disease	6 (6.52%)	4 (5.48%)	
Laboratory, median (IQR)			
Platelet , 10^9^/L	180.50 (132.50-242.75)	205.00 (177.00-247.00)	**0.046**
PT, seconds	13.05 (12.4-13.98)	12.80 (12.45-13.2)	0.074
APTT, seconds	39.15 (35.13-42.23)	38.5 (34.00-41.00)	0.132
D-dimer, ng/mL FEU	0.86 (0.35-1.65)	0.27 (0.17-0.60)	**0.000**
FDP, mg/dL	2.43 (1.43-4.7)	1.24 (0.785-2.09)	**0.000**
CRP, mg/L	7.31 (3.70-29.12)	3.96 (2.16-8.62)	**0.000**
Anticoagulant use, n (%)			
No anticoagulant	74 (80.4%)	71 (97.3%)	
Standard prophylaxis	9 (9.8%)	0 (0%)	
Therapeutic anticoagulant	9 (9.8%)	2 (2.7%)	
Venous thrombosis, n (%)			
VTE	9 (9.8%)	2 (2.7%)	0.114
NO	83 (90.2%)	71 (97.3%)	

*P* < 0.05 indicates that there is significant difference.

In this study, a retrospective analysis was made on the incidence of thrombosis and related laboratory data, including PLT, PT, APTT, DD, FDP and CRP. In the cancer and COVID-19 cohort, 9 patients (9.8%) received therapeutic anticoagulant therapy during hospitalization because of deep venous thrombosis of lower extremities, 9 patients (9.8%) received prophylactic anticoagulant therapy, where no thrombus occurred. Enoxaparin is selected for anticoagulant drugs, with no obvious adverse reactions found. There was no bleeding event among all COVID-19 patients. It was found that the levels of D-dimer, FDP and CRP in cancer and COVID-19 patients were significantly higher than those in non-cancer cohorts(*P*<0.05). There was no statistical difference about PT and APTT between them. There were 9 (9.8%) cancer and COVID-19 patients and 2 (2.7%) non-cancer patients found VTE during hospitalization. There was no significant difference in the incidence of thrombosis by Chi-square test for pairwise comparison (*P*=0.114).

There were 92 patients with both cancer and COVID-19 in the cohort (**Table 2**). The highest rates were lung cancer (28.26%), followed by gastrointestinal cancer (14.13%), liver cancer (9.78%), genitourinary cancer (7.61%), head and neck tumors (5.43%) and hematological malignant tumors or other (27.17%). Metastatic cancer accounted for 43.18% and 85.87% of the patients had received antineoplastic therapy. Besides, 64.28% of COVID-19 patients totally have been vaccinated, which may also be related to mild symptoms and reduced risk of complications.

**Table 2:** Distribution of cancer types among patients with COVID-19.

Site Of Cancer	
Lung	26 (28.26%)
Gastrointestinal	13 (14.13%)
Head and neck	5 (5.43%)
Liver	6 (9.78%)
Gynecologic malignancy	7 (7.61%)
Pancreas	5 (5.43%)
Gallbladder	5 (5.43%)
Other and hematology	25 (27.17%)
Metastatic cancer	40 (43.48%)

**Figure 2:**
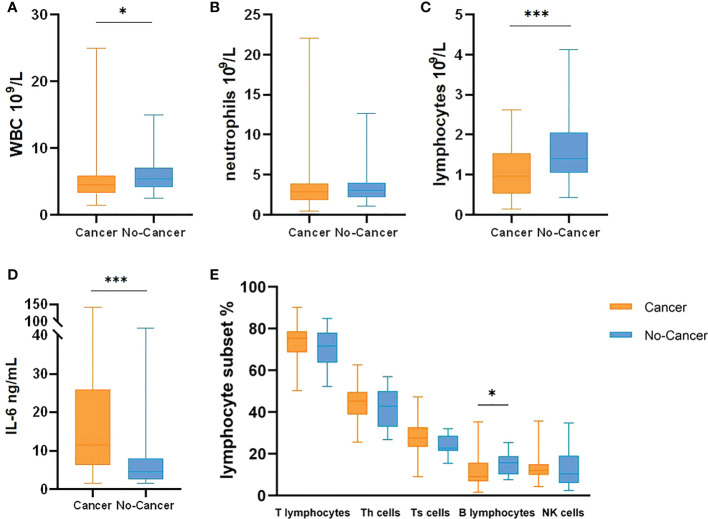
Leukocyte, IL-6 and Lymphocyte Subsets comparison between cancer patients with COVID-19 and no-cancer patients (**(A)** WBC, **(B)** neutrophils, **(C)** lymphocytes, **(D)** IL-6, **(E)** Lymphocyte Subsets]. *p < 0.05, ***p < 0.001.

At the same time, IL-6, white blood cells, neutrophils, lymphocytes, monocytes, CD3.CD45+T lymphocytes, CD3.CD4.CD45+ helper T cells, CD3.CD8.CD45+ suppression T cells, CD19.CD45+B lymphocytes and CD16.CD56.CD45+ natural killer cells (NK cells) were counted in the two cohorts (**Table 3**, **Figure 2**). The results showed that WBC, lymphocytes and B lymphocytes in cancer patients with COVID-19 were significantly lower than those in non-cancer group(*P*<0.05), but there was no significant difference in other lymphocyte subsets(*P*>0.05). IL-6 was significantly higher, contrasted by the non-cancer cohort (*P*< 0.05).

**Table 3:** Leukocyte and Lymphocyte Subpopulations of cancer patients with COVID-19 and COVID-19 patients.

	Cancer+Covid-19	Covid-19	*P*
Laboratory (median, IQR)			
WBC, 10^9^/L	5.72 (4.34-7.38)	6.04 (4.84-7.69)	**0.006**
IL-6, ng/mL	10.5 (5.95-18.8)	3.40 (2.08-6.38)	**0.000**
neutrophils, 10^9^/L	3.94 (2.54-4.67)	3.23 (2.54-4.11)	0.192
lymphocytes, 10^9^/L	1.40 (0.86-1.83)	1.94 (1.32-2.62)	**0.000**
monocytes, 10^9^/L	0.57 (0.42-0.66)	0.51 (0.38-0.74)	0.547
T lymphocytes CD3.CD45, %	75.32 (68.69-78.70)	71.66 (64.09-77.99)	0.317
Th cells CD3.CD4.CD45, %	45.18 (39.24-48.97)	42.73 (33.37-49.47)	0.491
Ts cells CD3.CD8.CD45, %	27.52 (23.31-32.11)	22.71 (21.50-28.43)	0.095
B lymphocytes CD19.CD45, %	9.08 (6.82-14.56)	15.68 (10.65-18.84)	**0.017**
NK cells CD16.CD56.CD45, %	12.10 (9.90-14.96)	10.37 (6.07-18.38)	0.420

*P* < 0.05 indicates that there is significant difference.

## Discussion

COVID-19 ‘s associated coagulation disorder was characterized by increased levels of D-dimer and fibrinogen, and slight abnormalities in prothrombin time, activated partial thromboplastin time and platelet count at the initial stage of infection (9, 10). Cancer is an established risk factor for thrombosis and bleeding during hospitalization, but it is not clear whether COVID-19 will further amplify these risks. Patell et al (11) found that the incidence of thrombosis was very high in hospitalized COVID-19 patients, with a 28-day incidence of 18% in non-cancer patients and 14% in cancer patients. It may reflect the overwhelming thrombosis inflammatory state of COVID-19, which overshadowed the hypercoagulable state of active cancer. However, in COVID-19 patients of this study, the incidence of thrombosis in cancer patients was 9.8%, and 2.7% in the non-cancer cohort. It was considered that the virulence of the SARS-CoV-2 Omicron BA.2 variant was weakened and the symptoms were mild. Although the results are not statistically difference, it may be because the base of non-cancer COVID-19 patients in the treatment center is not large enough and follow-up time is short, which limits the inference. Some studies have pointed out that out-of-hospital thrombotic events still occur in patients with new coronary pneumonia (12). The average length of hospital stay for these patients was two weeks, which was so short that we missed out-of-hospital thrombosis. Therefore, we need long-term follow-up of these patients. The inflammatory state caused by COVID-19 added the hypercoagulable state of cancer may cause thrombosis to occur more easily (13). Although observational studies have shown benefits of anticoagulation, the rigorous evidence to establish an optimal anticoagulation regimen in patients with COVID-19 is lack. Even with multiple clinical studies, there is still no consensus on the choice of prophylactic and therapeutic doses of anticoagulation. Guidelines recommend primary prophylactic anticoagulant therapy for COVID-19 patients with locally advanced or metastatic pancreatic cancer (6). Combined with this study, primary prophylactic anticoagulant therapy is recommended for advanced cancer patients with high risk factors (increased DD, FDP, CRP), such as low molecular weight heparin.

SARS-CoV-2 enters host cells by interacting with angiotensin converting enzyme 2 (ACE2) receptors (14), through bringing about endothelial cell injury and endodermatitis, regulation of angiotensin converting enzyme 2 receptor, and activation of blood coagulation system, resulting in microthrombus deposition and microvascular dysfunction (15–18). Monocyte activation can be involved in thrombotic events by stimulating cytokine release or leading endotheliitis (12). A meta-analysis of multiple clinical studies found that the combined incidence of venous thromboembolism and pulmonary embolism during hospitalization in patients with new coronary pneumonia was 17% and 7.1%, respectively, while the incidence of major bleeding was 3.9%. The incidence of thromboembolism was higher in critically ill patients (27.9%), but the rate of bleeding was higher in patients receiving anticoagulation (21.4%) (19). Katsoularis et al (20) found that pulmonary vascular embolism also accounted for a large proportion of thrombosis, but no pulmonary embolism was found in our team, which may be related to the weakening of pathogenicity of mutants. Prolonged PT and APTT can be seen in this queue, and the increased risk of bleeding may be related to endothelial dysfunction, coagulation disorder or disseminated intravascular coagulation (21). It was needed to pay attention to the risk of bleeding events.

As the recent CDC update, subvariants BA.2, BA.4, BA.5 and BA.2.12. have been found at present. The transmission power of BA.2 is at least 30% higher than that of BA.1. The transmission power of BA.2.12.1 in the United States is estimated to be 23% to 27% higher than that of its predecessor BA.2. A study in South Africa showed that BA.4 and BA.5 variants could reduce neutralizing antibody titers in serum samples of patients who had been infected with the original Omicron virus by nearly 88% and those who had been vaccinated by 67% (22). The enhancement of the transmission ability of subvariants is related to the stronger escape of humoral immunity (22). This study also focused on the changes of immune system in COVID-19 patients with cancer, and compared IL-6, white blood cells and lymphocyte subsets. It was showed that IL-6 in cancer and COVID-19 cohort was higher than that in non-cancer cohort, but WBC, lymphocytes and B lymphocytes were decreased, and there was no significant difference in other lymphocyte subsets. Lymphopenia is a sign of impaired cellular immune function, which has been reported in 67-90% of COVID-19 patients (23–25). Studies have demonstrated that ACE2 receptors are expressed on the surface of lymphocytes, thereby SARS-CoV-2 may directly infect these cells and eventually lead to their dissolution (3). In addition, the characteristic of cytokine storm is significantly increased levels of interleukin (mainly IL-6, IL-2, IL-7, granulocyte colony stimulating factor, interferon-gamma inducible protein 10, monocyte chemoattractant protein-1) and tumor necrosis factor-α, which may promote lymphocyte apoptosis (26–28). This may be more prominent in cancer patients with complications, and it may also inhibit lymphocyte proliferation (29). A number of studies have indicated that lymphopenia in patients with COVID-19 is related to prognosis (30–32). In addition, neutropenia is less common but is also a negative prognostic marker (25, 31). Studies on specific lymphocyte subsets have shown that the decrease of CD4+T cell (33), CD8+T cell (29), B cell, NK cell and total lymphocyte count in peripheral blood is related to severe COVID-19 (34, 35). For cancer patients, the decrease of TBNK subsets cells is also associated with a poor prognosis (36, 37). Some studies have found that plasma cells are generally elevated in infection, and more activated B cells are involved in Covid-19 immunity (38). The reduction of B cells in this paper may be due to a decrease in the number of total lymphocytes, or the immune status of cancer patients. No changes in T cell subsets were detected in this paper, and the sample size can be expanded to continue analysis.

According to the interim guidelines of the International Society of Hemostasis and Thrombosis, it is recommended that laboratory counts are longitudinally evaluated during COVID-19 ‘s hospitalization, including leukocyte classification, D-dimer, prothrombin time, fibrinogen and so on (39).

## Conclusions

Although there was no significant difference in the incidence of thrombosis among Omicron BA.2 COVID-19 patients with cancer and COVID-19 group, the levels of D-dimer, FDP and CRP increased significantly. Prophylactic anticoagulation was found to be effective here, and prophylactic anticoagulation was recommended for cancer patients with high risk factors, while paying attention to the occurrence of bleeding events. When patients had both cancer and SARS-CoV-2, lymphopenia and decreased B lymphocytes becomes more pronounced, and IL-6 was also elevated. The detection of leukocyte classification, D-dimer, prothrombin time and fibrinogen at different time points are helpful for the diagnosis, anticoagulation and antitumor treatment of COVID-19 patients with cancer. Overall, Omicron BA.2 variant has strong transmission, mild symptoms and reduced risk of complications.

## Data availability statement

The raw data supporting the conclusions of this article will be made available by the authors, without undue reservation.

## Ethics statement

Written informed consent was obtained from the individual(s) for the publication of any potentially identifiable images or data included in this article.

## Author contributions

JY was responsible for manuscript analysis writing and revision. XC was responsible for idea design and treatment. NW, SW, YG, YZ, LZ were responsible for patients treatment and data collection. ZL was responsible for writing guidance and idea. All authors contributed to the article and approved the submitted version.

## Conflict of interest

The authors declare that the research was conducted in the absence of any commercial or financial relationships that could be construed as a potential conflict of interest.

## Publisher’s note

All claims expressed in this article are solely those of the authors and do not necessarily represent those of their affiliated organizations, or those of the publisher, the editors and the reviewers. Any product that may be evaluated in this article, or claim that may be made by its manufacturer, is not guaranteed or endorsed by the publisher.

## Abbreviations

PLT, Platelet; PT, Prothrombin Time; APTT, Active Partial Thromboplasting Time; DD, D-dimer; FDP, Fibrin/Fibrinogen Degradation Products; CRP, C-reactive protein; IL-6, Interleukin- 6.
